# Gut microbial beta-glucuronidase and glycerol/diol dehydratase activity contribute to dietary heterocyclic amine biotransformation

**DOI:** 10.1186/s12866-019-1483-x

**Published:** 2019-05-16

**Authors:** Jianbo Zhang, Christophe Lacroix, Esther Wortmann, Hans-Joachim Ruscheweyh, Shinichi Sunagawa, Shana J. Sturla, Clarissa Schwab

**Affiliations:** 1 Department of Health Sciences and Technology, ETH Zürich, Zürich, Switzerland; 2 Department of Biology, ETH Zürich, Zürich, Switzerland; 3 Present Address: Department of Biological Engineering, Massachusetts Institute of Technology, Cambridge, MA USA

**Keywords:** Heterocyclic amines (HCA), Glycerol/diol dehydratase, Reuterin, *β*-Glucuronidase, *Faecalibacterium prausnitzii*, *Eubacterium hallii*

## Abstract

**Background:**

Consuming red and processed meat has been associated with an increased risk of colorectal cancer (CRC), which is partly attributed to exposure to carcinogens such as heterocyclic amines (HCA) formed during cooking and preservation processes. The interaction of gut microbes and HCA can result in altered bioactivities and it has been shown previously that human gut microbiota can transform mutagenic HCA to a glycerol conjugate with reduced mutagenic potential. However, the major form of HCA in the colon are glucuronides (HCA-G) and it is not known whether these metabolites, via stepwise microbial hydrolysis and acrolein conjugation, are viable precursors for glycerol conjugated metabolites. We hypothesized that such a process could be concurrently catalyzed by bacterial beta-glucuronidase (B-GUS) and glycerol/diol dehydratase (GDH) activity. We therefore investigated how the HCA-G PhIP-N2-β-D-glucuronide (PhIP-G), a representative liver metabolite of PhIP (2-Amino-1-methyl-6-phenylimidazo [4,5-*b*] pyridine), which is the most abundant carcinogenic HCA in well-cooked meat, is transformed by enzymatic activity of human gut microbial representatives of the phyla *Firmicutes*, *Bacteroidetes*, and *Proteobacteria*.

**Results:**

We employed a combination of growth and enzymatic assays, and a bioanalysis approach combined with metagenomics*.* B-GUS of *Faecalibacterium prausnitzii* converted PhIP-G to PhIP and GDH of *Flavonifractor plautii*, *Blautia obeum*, *Eubacterium hallii*, and *Lactobacillus reuteri* converted PhIP to PhIP-M1 in the presence of glycerol. In addition, B-GUS- and GDH-positive bacteria cooperatively converted PhIP-G to PhIP-M1. A screen of genes encoding B-GUS and GDH was performed for fecal microbiome data from healthy individuals (*n* = 103) and from CRC patients (*n* = 53), which revealed a decrease in abundance of taxa with confirmed GDH and HCA transformation activity in CRC patients.

**Conclusions:**

This study for the first time demonstrates that gut microbes mediate the stepwise transformation of PhIP-G to PhIP-M1 via the intermediate production of PhIP. Findings from this study suggest that targeted manipulation with gut microbes bearing specific functions, or dietary glycerol supplementation might modify gut microbial activity to reduce HCA-induced CRC risk.

**Electronic supplementary material:**

The online version of this article (10.1186/s12866-019-1483-x) contains supplementary material, which is available to authorized users.

## Background

**Fig. 1:**
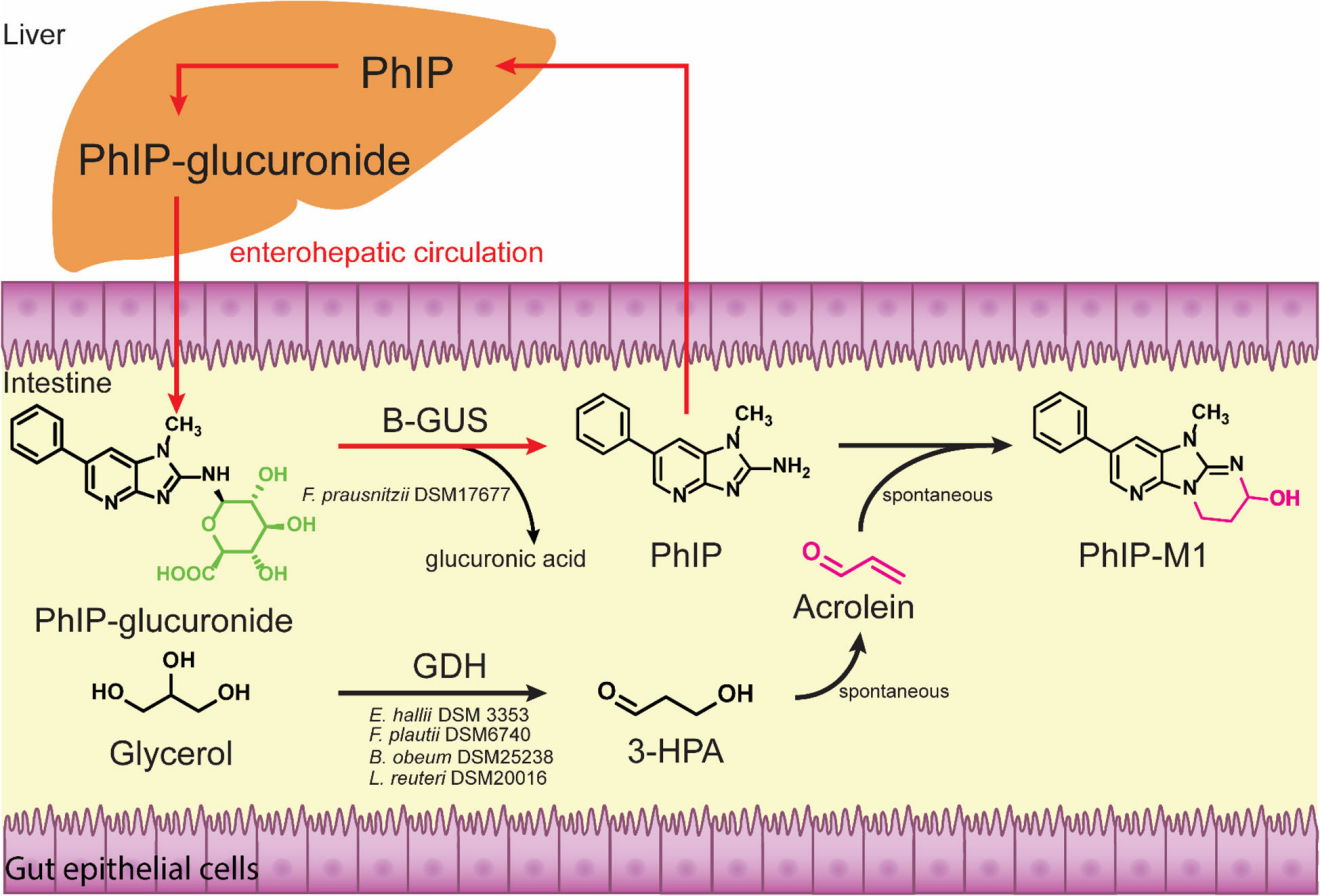
Bacterial activities contributing to activation and detoxification processes governing the enterohepatic disposition of PhIP. B-GUS, *β*-glucuronidase; GDH, glycerol/diol dehydratase; 3-HPA, 3-hydroxypropionaldehyde

Regular consumption of cooked and processed meat increases the risk for colorectal cancer (CRC) due to the prolonged exposure to meat-derived carcinogens such as 2-amino-1-methyl-6-phenylimidazo [4,5-*b*] pyridine (PhIP), 2-amino-3,8-dimethylimidazo [4,5-*f*]quinoxaline (MeIQx) and other heterocyclic amines (HCA) [1, 2]. After being absorbed in the gut, HCA are activated by liver cytochrome P450 enzymes and *N*-acetyltransferase or sulfotransferase to form acetyl esters or sulfates [3, 4]. These conjugates are not stable and decompose to nitrenium intermediates, which can form DNA adducts [5], induce DNA mutation in bacterial and mammalian cell-based genotoxicity assays [6], and induce tumours in the large intestine of rats [7]. Alternatively, *N*-glucuronidation at the *N*^2^- or 3-positions of HCA or OH-*N*-HCA, mainly catalysed by uridine diphosphate (UDP)–glucuronosyltransferases in the liver, competes with the activation pathway and results in the formation of inactive glucuronide conjugates including HCA-*N*^2^-*β*-D-glucuronide (HCA-G), HCA-3-*β*-D-glucuronide, OH-*N*-HCA-G, and OH-*N*-HCA-3-*β*-D-glucuronide [8–11]. Of these metabolites, *N*^2^-glucuronide conjugates of HCA and OH-*N*-HCA are the major metabolites in human hepatocytes, accounting for up to 71% of the HCA dose [12]. These glucuronide conjugates, together with the unchanged HCA, enter the urine or colon with the bile (major route) (Fig. 1) [13].

In the intestine, glucuronide conjugates can be hydrolyzed by bacterial beta-glucuronidases (B-GUS, EC 3.2.1.31), which liberate potentially bioactive aglycones [14]; the release of OH-*N*-products could potentially lead to mutagenicity via interaction with colon epithelial cells. Additionally, for HCA-G, HCA may be taken up into the liver, where it may be activated and potentially damage molecular targets such as DNA, or it may be converted back to HCA-G, re-entering the intestine (Fig. 1), while contributing to enterohepatic circulation which can prolong the duration of HCA exposure. Indeed, there was a second peak of PhIP and its liver metabolites in fecal and urine samples of human subjects 48–72 h after ingesting well-cooked chicken containing 0.9–5 μg PhIP [15].

While the capacity of human gut microbiota to hydrolyze HCA-G is poorly understood, there are several studies supporting that human gut microbiota can transform HCA to glycerol conjugates. For example, formation of the PhIP microbial metabolite 7-hydroxy-5-methyl-3-phenyl-6,7,8,9-tetrahydropyrido [3’,2’:4,5]imidazo [1,2-*a*]pyrimidin-5-ium chloride (PhIP-M1) has been observed during growth of the gut microbes *Lactobacillus reuteri*, *Eubacterium hallii*, and strains of *Enterococcus* in the presence of glycerol (Fig. 1) [16–19]. The formation of HCA-M1 from PhIP involves a multi-step process: (1) the enzymatic reduction of glycerol to 3-hydroxy-propionaldehyde (3-HPA) by coenzyme B_12_-dependent glycerol/diol dehydratases (GDH, EC 4.2.1.28 and EC 4.2.1.30), (2) the accumulation of 3-HPA, (3) spontaneous dehydration of 3-HPA to form acrolein, and (4) the chemical reaction of acrolein with HCA [18]. Consistent with the glycerol conjugation reaction blocking the primary amino group of HCA, PhIP-M1 and 9-hydroxyl-2,7-dimethyl-7,9,10,11-tetrahydropyrimido[20,10:2,3] imidazo [4,5-*f*]quinoxaline (MeIQx-M1) have lower mutagenicity than PhIP and MeIQx in the Ames test with S9 activation [15, 20] and were not observed to induce cell malignant transformation of BALB/c 3 T3 cells [20, 21]. Some strains further metabolize 3-HPA to 1,3-propanediol (1,3-PD) [22]. A second substrate of GDH is 1,2-propanediol (1,2-PD), a fermentation product of fucose and rhamnose [22, 23]. GDH reduces 1,2-PD to propionaldehyde, which can be further metabolized to propionate and 1-propanol as end products [22, 24].

Given that B-GUS is a regular enzymatic activity of gut microbiota [14], and that gut microbes that possess GDH can convert HCA to HCA-M1, we hypothesized that HCA-G hydrolysis and glycerol-dependent HCA conjugation catalyzed by bacterial GDH and B-GUS concurrently occur (Fig. 1). We tested this hypothesis by performing co-culture fermentations with B-GUS and GDH positive gut microbes using PhIP-*N*^2^-*β*-D-glucuronide (PhIP-G) as a representative HCA-glucuronide. PhIP-G is the second most abundant *N*-linked PhIP-glucuronide in humans [12]. Compared to OH-*N*^2^-PhIP glucuronide conjugates, the lack of hydroxyl moiety at the exocyclic amino group yields a primary amine after de-conjugation. This primary amine is key for glycerol conjugation [18], therefore, PhIP-G is an appropriate model metabolite for the stepwise reaction by B-GUS and GDH positive strains. As HCA intake has been linked to CRC, we investigated the potential of fecal microbiomes to release PhIP from PhIP-G and convert it to HCA-M1. Metagenomes (*n* = 156) of healthy and CRC patients [25] were screened for gene abundance and contributing taxa of *b-gus* and *gdh*. The results provide a mechanistic model of how gut microbiota might influence PhIP disposition and modulate carcinogenesis risk.

## Results

### Strain selection

We aimed to investigate GDH and B-GUS activity of gut microbes in single and co-cultures. Representative strains of species predicted to possess GDH activity were chosen based on a previous study, which used fecal metagenomes to identify species harboring *gdh* [24], Table 1). Gut microbes harboring *b-gus* were identified by Dabek et al. [26]. All strains were tested for both GDH and B-GUS activity.

**Table 1:** Strains used, and presence of glycerol/diol dehydratase (GDH) and *β*–glucuronidase (B-GUS) encoding genes. The presence of *gdh* was predicted by metagenome analysis of human feces [24] and was confirmed for the used strains based on genome analysis (https://www.ncbi.nlm.nih.gov/genome/microbes/). The presence of *b-gus* was predicted by Dabek et al. [26] and McIntosh et al. [27]

Strain name	Strain ID	*gdh*	*b-gus*
*Blautia obeum*	DSM 25238	+	–
*Eubacterium eligens*	DSM 3376	–	+
*Eubacterium hallii*	DSM 3353	+	–
*Faecalibacterium prausnitzii*	DSM 17677	–	+
*Flavonifractor plautii*	DSM 6740	+	–
*Intestinimonas butyriciproducens*	DSM 26588	+	–
*Lactobacillus reuteri*	DSM 20016	+	–
*Roseburia hominis*	DSM 16839	–	+
*Roseburia intestinalis*	DSM 14610	–	+
*Ruminococcus gnavus*	ATCC 29149	+	–
*Veillonella dispar*	ATCC 17748	+	–
*Bacteroides fragilis*	ATCC 25285	–	+
*Citrobacter freundii*	CB 36	+	–
*Klebsiella pneumoniae*	CB 35	+	–

+/− gene encoding the indicated enzyme is present/absent in the representative genome. All strains are commercially available except *C. freundii* and *K. pneumoniae*, which were obtained in-house collection of Laboratory of Food Biotechnology, ETH Zurich

### Strains with active GDH

**Fig. 2:**
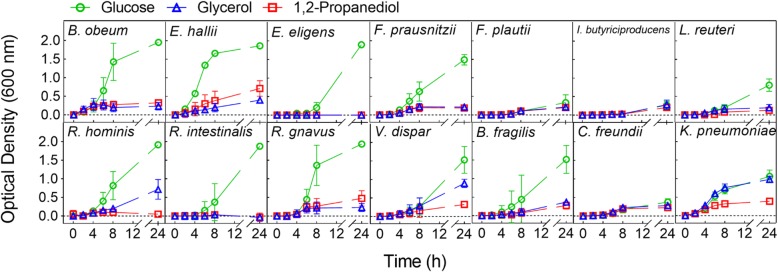
Bacterial growth in the presence of glucose, glycerol, and 1,2-propanediol. Strains were grown in YCFA medium supplied with glucose (green circle), glycerol (blue triangle) or 1,2-propanediol (red rectangle) (each added at 50 mM) at 37 °C for 24 h. Optical density (OD) was measured at 600 nm

To determine strains with active GDH, growth capacity and metabolic activity of the selected strains (Table 1) were assessed. Incubations were performed in anaerobically prepared yeast-casitone-fatty acid (YCFA) [28] medium at 37 °C in the presence of glycerol or 1,2-PD (50 mM), or glucose (50 mM) as a control. We previously showed that the presence of active GDH leads to both formation of propionate and 3-HPA from 1,2-PD and glycerol, respectively, during growth [24]. Thus, substrate utilization and major metabolite production (i.e. formate, acetate, propionate, lactate, butyrate, and 1,3-PD) were determined using high pressure liquid chromatography with a refractive index detector (HPLC-RI). All strains grew in the presence of glucose and utilized the provided substrate with the exception of *Intestinimonas butyriciproducens,* which nonetheless produced butyrate (Fig. 2, Additional file 1: Table S1). Moreover, six strains, i.e. *L. reuteri*, *E. hallii*, *Blautia obeum*, *Flavonifractor plautii*, *Ruminococcus gnavus*, and *Klebsiella pneumoniae* were found to also grow in the presence of 1,2-PD (Fig. 2) and to produce propionate (Table 2). *E. hallii*, *F. plautii*, and *K. pneumoniae* used significantly (*p* < 0.05) more 1,2-PD and produced significantly (*p* < 0.05) higher amounts of propionate compared to the other strains (Table 2). Other fermentation metabolites formed were acetate (0.8–9.5 mM, by *B. obeum*, *F. plautii*, *R. gnavus*, and *K. pneumoniae*), formate (1.0–5.3 mM, by *E. hallii*, *R. gnavus*, and *K. pneumoniae*), and butyrate (4.1–4.3 mM, by *E. hallii* and *F. plautii*). Of the six strains discovered to use 1,2-PD, five strains, i.e. *B. obeum*, *E. hallii*, *F. plautii*, *L. reuteri*, and *K. pneumoniae*, also metabolized glycerol. Finally, four of these, i.e. *K. pneumoniae*, *E. hallii*, *F. plautii*, and *L. reuteri*, produced 1,3-PD (Table 2). Glycerol consumption and 1,3-PD formation were significantly (*p* < 0.05) higher for *K. pneumoniae.* Other metabolites produced were formate (5.3–17.5 mM, by *K. pneumoniae*), acetate (5.2–10.7 mM, by *F. plautii* and *K. pneumoniae*), and butyrate (4.3–7.8 mM, by *F. plautii*) (Table 2). *Veillonella dispar* and *Roseburia hominis* used glycerol, but produced mainly formate and acetate, and no 1,3-PD (Fig. 2 and Table 2). *Citrobacter freundii* did not use the carbon substrates supplied and likely formed formate and acetate from other components of the YCFA medium (Additional file 1: Table S1). These results suggest that six strains, i.e. *B. obeum, E. hallii*, *F. plautii*, *L. reuteri*, *R. gnavus*, and *K. pneumoniae*, were able to catalyze the reduction of glycerol/1,2-PD, presumably mediated by GDH, and were therefore examined further for their capacity to convert the mutagenic PhIP to a non-mutagenic PhIP-M1 [16].

**Table 2:** Substrate utilization and metabolite production of single strains in the presence of glycerol or 1,2-propanediol. Strains were grown in anaerobic YCFA supplied with glycerol or 1,2-propanediol (1,2-PD) (both 50 mM) at 37 °C for 24 h. *E. eligens* did not grow and utilize glycerol and 1,2-PD. n.d. = not determined; *n* = number of biological replicates. Values are presented as mean ± standard deviation

Strain^a^	Substrate	n	Substrate utilization (mM)	Metabolite production (mM)				
				Formate	Acetate	Propionate	Butyrate	1,3-PD
*B. obeum*	1,2-PD	4	−17.9 ± 4.3^A*^	0^A^	1.5 ± 1.7^AB^	5.2 ± 0.4^A^	− 0.2 ± 0.6^A^	n.d.
	glycerol	6	−2.1 ± 1.2^a^	0^a^	1.5 ± 1.4^a^	− 0.6 ± 0.4^a^	0^a^	0
*E. hallii*	1,2-PD	6	−46.6 ± 6.4^B^	1.0 ± 0.8^A^	− 1.6 ± 7.8^A^	19.3 ± 8.0^B^	4.3 ± 0.7^B^	n.d.
	glycerol	6	− 13.3 ± 3.0^b^	0.7 ± 1.0^b^	− 4.6 ± 2.5^b^	0 ± 0.8^a^	0 ± 0.8^a^	1.9 ± 0.7^a^
*F. prausnitzii*	1,2-PD	2	− 0.7^C^	0^A^	−1.4 ^A^	0^C^	1.5^A^	n.d.
	glycerol	3	−1.6 ± 0.4^c^	0.9 ± 1.6^a^	1.3 ± 0.5^a^	0.3 ± 0.2^a^	0.3 ± 0.6^a^	0 ^a^
*F. plautii*	1,2-PD	4	−48.1 ± 1.2^B^	0^A^	2.2 ± 1.4^A^	11.6 ± 3.9^B^	4.1 ± 0.3^B^	n.d.
	glycerol	4	−9.1 ± 3.0^c^	0^a^	5.2 ± 0.7^c^	−0.5 ± 0.4^a^	4.3 ± 0.5^b^	1.2 ± 0.7^a^
*I. butyriciproduens*	1,2-PD	5	−1.2 ± 2.9^C^	0^A^	2.6 ± 2.6^A^	− 0.1 ± 0.5^C^	5.4 ± 0.6^B^	n.d.
	glycerol	3	− 0.8 ± 1.4^ac^	0^a^	2.5 ± 1.7^ac^	−0.1 ± 0.1^a^	5.3 ± 0.5^b^	3.0 ± 0.1^b^
*L. reuteri*	1,2-PD	3	−9.8 ± 4.2^C^	0^A^	−1.0 ± 1.1^A^	0.9 ± 0.5^C^	0^A^	n.d.
	glycerol	3	−14.4 ± 8.8^b^	0^a^	−0.4 ± 1.1^a^	0^a^	0^a^	5.2 ± 0^b^
*R. hominis*	1,2-PD	3	0^D^	0^A^	0^A^	0^C^	0^A^	n.g.
	glycerol	5	−5.9 ± 1.1^c^	0^a^	−8.5 ± 1.3^d^	−0.5 ± 0.2^a^	7.4 ± 1.6^b^	0
*R. intestinalis*	1,2-PD	3	−0.4 ± 0.4^C^	0^A^	− 0.4 ± 0.4^A^	−0.3 ± 0.1^C^	0.4 ± 0.2^A^	n.d.
	glycerol	3	−1.4 ± 0.6^ac^	0^a^	1.3 ± 1.0^a^	0.4 ± 0.4^a^	0.4 ± 0.2^a^	0^a^
*R. gnavus*	1,2-PD	3	−18.0 ± 5.1^A^	1.1 ± 1.9^A^	2.2 ± 1.4^AB^	7.9 ± 2.4^A^	0.2 ± 0.3^A^	n.d.
	glycerol	3	− 1.4 ± 1.2^a^	0^a^	0.8 ± 1.4^ae^	0^a^	0^a^	0.2 ± 0.3^a^
*V. dispar*	1,2-PD	4	−1.7 ± 2.4^D^	1.4 ± 1.6^A^	0 ± 2.3^AB^	0.6 ± 0.8^C^	0^A^	n.d.
	glycerol	4	−4.4 ± 1.4^ac^	6.6 ± 1.2^c^	−2.4 ± 1.9^be^	− 0.5 ± 0.5^a^	−0.2 ± 0.4^a^	0^a^
*B. fragilis*	1,2-PD	4	−1.0 ± 1.4^C^	0^A^	−0.1 ± 1.6^A^	0.3 ± 0.5^C^	−0.4 ± 0.4^A^	n.d.
	glycerol	3	− 1.0 ± 0.9^ac^	0^a^	0 ± 0.5^a^	0.6 ± 0.6^a^	0^a^	0^a^
*C. freundii*	1,2-PD	3	−1.4 ± 1.8^D^	5.4 ± 0.6^B^	7.2 ± 1.2^B^	1.5 ± 0.6^D^	0^A^	n.d.
	glycerol	3	−2.1 ± 3.2^a^	6.0 ± 0.5^c^	5.3 ± 2.0^c^	−0.8 ± 0.8^a^	0^a^	0^a^
*K. pneumoniae*	1,2-PD	3	− 42.7 ± 1.1^B^	5.3 ± 0.1^B^	3.1 ± 1.5^AB^	16.7 ± 1.0^B^	0^A^	n.d.
	glycerol	3	− 48.7 ± 0.8^d^	17.5 ± 1.2^d^	10.7 ± 0.2^f^	0^a^	0^a^	16.4 ± 3.3^c^

*Different letters represent the significant differences (*p* < 0.05) on the substrate utilization or metabolite production between different strains using multiple comparisons with 2-way ANOVA. Capital letters are for 1,2-PD and the corresponding metabolites, small letters are for glycerol and the corresponding metabolites

### GDH-positive bacteria convert PhIP to PhIP-M1

**Fig. 3:**
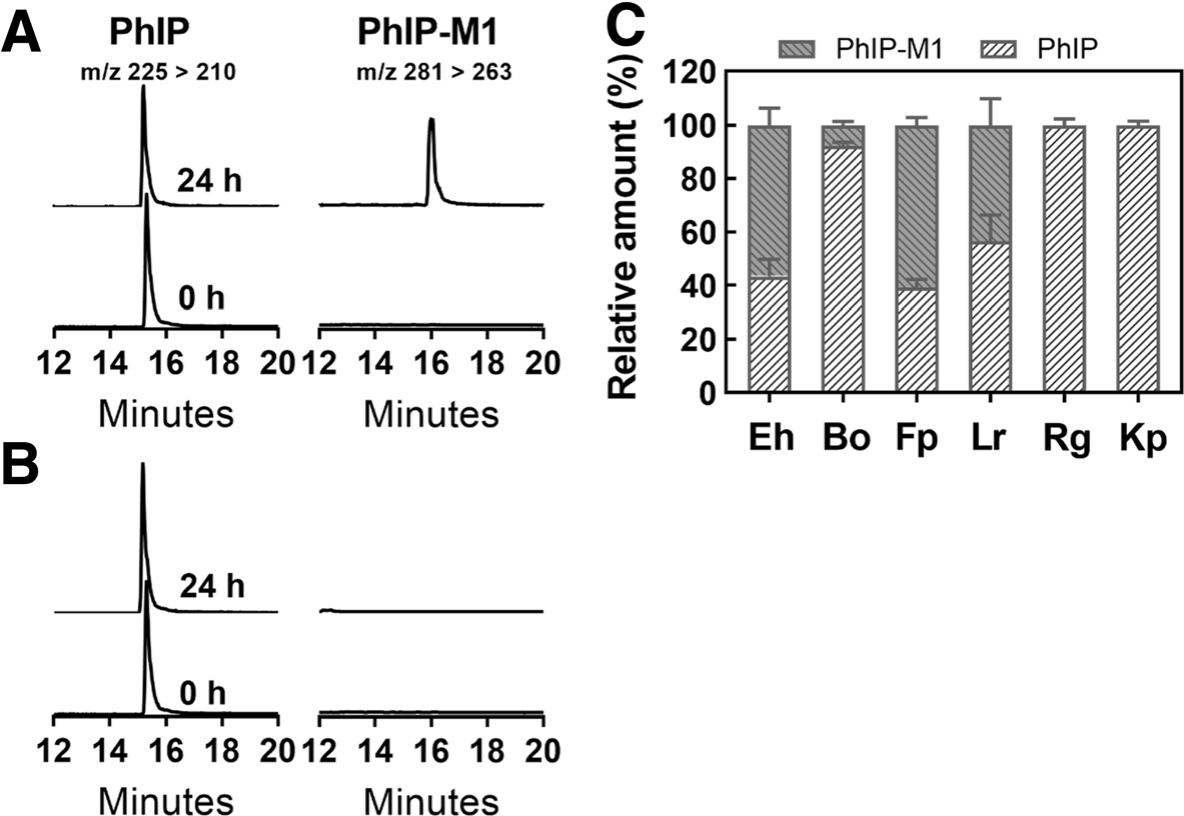
Conversion of PhIP to PhIP-M1 by glycerol/1,2-propanediol utilizing bacteria during growth in YCFA. Representative ion chromatograms of PhIP and its metabolites PhIP-M1 during the growth of **a** *F. plautii* or **b** *R. gnavus* immediately (0 h) or 24 h after inoculation. **c** Relative amount of PhIP and its metabolite PhIP-M1 during growth of: Eh. *E. hallii* (*n* = 3), Bo. *B. obeum* (*n* = 5), Fp. *F. plautii* (*n* = 4), Lr. *L. reuteri* (*n* = 3), Rg. *R. gnavus* (*n* = 5), and Kp. *K. pneumoniae* (*n* = 2) in YCFA with 50 mM glycerol and PhIP (200 nM) at 37 °C for 24 h. PhIP and PhIP-M1 were analysed using nanoLC-ESI-MS^2^. Main transitions are indicated

To investigate whether bacteria that possess GDH activity are able to mediate PhIP to PhIP-M1 conversion, *B. obeum, F. plautii*, *R. gnavus*, and *K. pneumoniae* were incubated with 200 nM PhIP in YCFA medium in the presence of 50 mM glycerol. *E. hallii* and *L. reuteri* were used as positive controls. Levels of PhIP and PhIP-M1 were monitored using nano flow liquid chromatography electrospray ionization tandem mass spectrometry (nanoLC-ESI-MS^2^). As anticipated, *E. hallii* and *L. reuteri* converted PhIP to PhIP-M1, and *F. plautii* and *B. obeum* were newly identified as having the capacity to convert PhIP to PhIP-M1 (Fig. 3). For example, a LC-MS peak corresponding to PhIP-M1 appeared after 24 h incubation of PhIP with *F. plautii* (Fig. 3a, Additional file 1: Figure S1), but not for *R. gnavus* (Fig. 3b) or *K. pneumoniae* (data not shown). *F. plautii* had a similar transformation efficiency to *L. reuteri* and *E. hallii* (43–60% PhIP was converted to PhIP-M1 in 24 h), which was higher than that of *B. obeum* (8 ± 1%) (Fig. 3c). These results indicate that not all GDH positive strains release sufficient amounts of acrolein to transform PhIP.

### B-GUS-positive bacteria actively convert PhIP-*N*^*2*^-*β*-D-glucuronide (PhIP-G) to PhIP and/or PhIP-M1

Having established the capacity of known and new human gut microbes with active GDH to convert PhIP to PhIP-M1, we addressed whether PhIP-G could be accessible to selected gut microbes by the action of B-GUS, considering its presence in the gut being largely in glucuronidated form. We confirmed that *Faecalibacterium prausnitzii*, *R. hominis*, and *Roseburia intestinalis* strains previously reported to have B-GUS activity [26], were active in our hands by means of a colorimetric assay with *p*-nitrophenol-*O*-*β*-D-glucuronide (PNP-G) as a substrate. Crude cell extracts of overnight cultures of *F. prausnitzii* (0.73 ± 0.21 U mg^− 1^ protein) had higher B-GUS activities than those of *R. hominis* and *R. intestinalis* (0.09–0.17 U mg^− 1^ protein) (Additional file 1: Table S2). *Eubacterium eligens* had very low B-GUS activity (0.02 ± 0.00 U mg^− 1^ protein), and all of the other strains were B-GUS negative and not further tested (Additional file 1: Table S2). We additionally performed alignments of putative proteins of *E. hallii* DSM 3353 against the B-GUS databases provided by McIntosh et al. [27] and Pollet et al. [29] but did not find any significant matches in the databases which would indicate the presence of putative B-GUS proteins encoded by the genome of *E. hallii*.

**Fig. 4:**
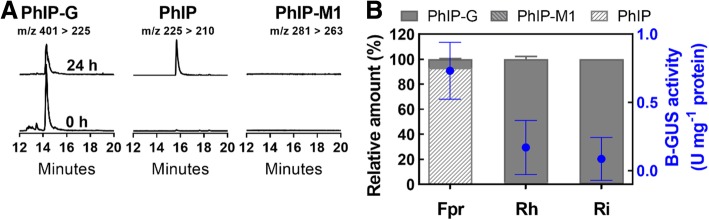
Transformation of PhIP-*N*^2^-*β*-D-glucuronide (PhIP-G) by *β*-glucuronidase (B-GUS) positive strains during growth in YCFA. Representative chromatographs of PhIP-G and its metabolites PhIP or PhIP-M1 during growth of (A) *F. prausnitzii*. (B) Relative amount of PhIP-G, PhIP and PhIP-M1 during growth of: Fpr. *F. prausnitzii* (*n* = 6), Rh. *R. hominis* (*n* = 4), and Ri. *R. intestinalis* (*n* = 2) in YCFA with glycerol and glucose (50 mM) and PhIP-G (200 nM) at 37 °C for 24 h. PhIP-G, PhIP, and PhIP-M1 were analysed using nanoLC-ESI-MS^2^. The B-GUS activity (blue circle) of the bacterial cell extracts was tested with PNP-G. Main transitions are indicated

To identify whether strains of *F. prausnitzii*, *R. hominis*, and *R. intestinalis* hydrolyzed PhIP-G, overnight cultures were incubated in YCFA supplied with 200 nM PhIP-G and 50 mM glycerol and glucose at 37 °C for 24 h. *F. prausnitzii* but not *R. hominis* and *R. intestinalis,* converted 93 ± 0.4% of PhIP-G to PhIP during growth (Fig. 4a, c and Additional file 1: Figure S2).

### B-GUS-positive and GDH-positive bacteria cooperatively convert PhIP-G to PhIP-M1

To test if PhIP-G can be converted to PhIP-M1 in the presence of B-GUS and GDH, we investigated co-cultures of B-GUS-positive *F. prausnitzii* and *E. eligens,* and of GDH-positive *F. plautii, L. reuteri*, and *B. obeum.* Growth in YCFA with 50 mM glycerol, 50 mM glucose and 200 nM PhIP-G at 37 °C for 24 h was determined using quantitative PCR (qPCR) and primers listed in Additional file 1: Table S3. Levels of PhIP-G, PhIP, and PhIP-M1 were monitored using (nanoLC-ESI-MS^2^).

**Fig. 5:**
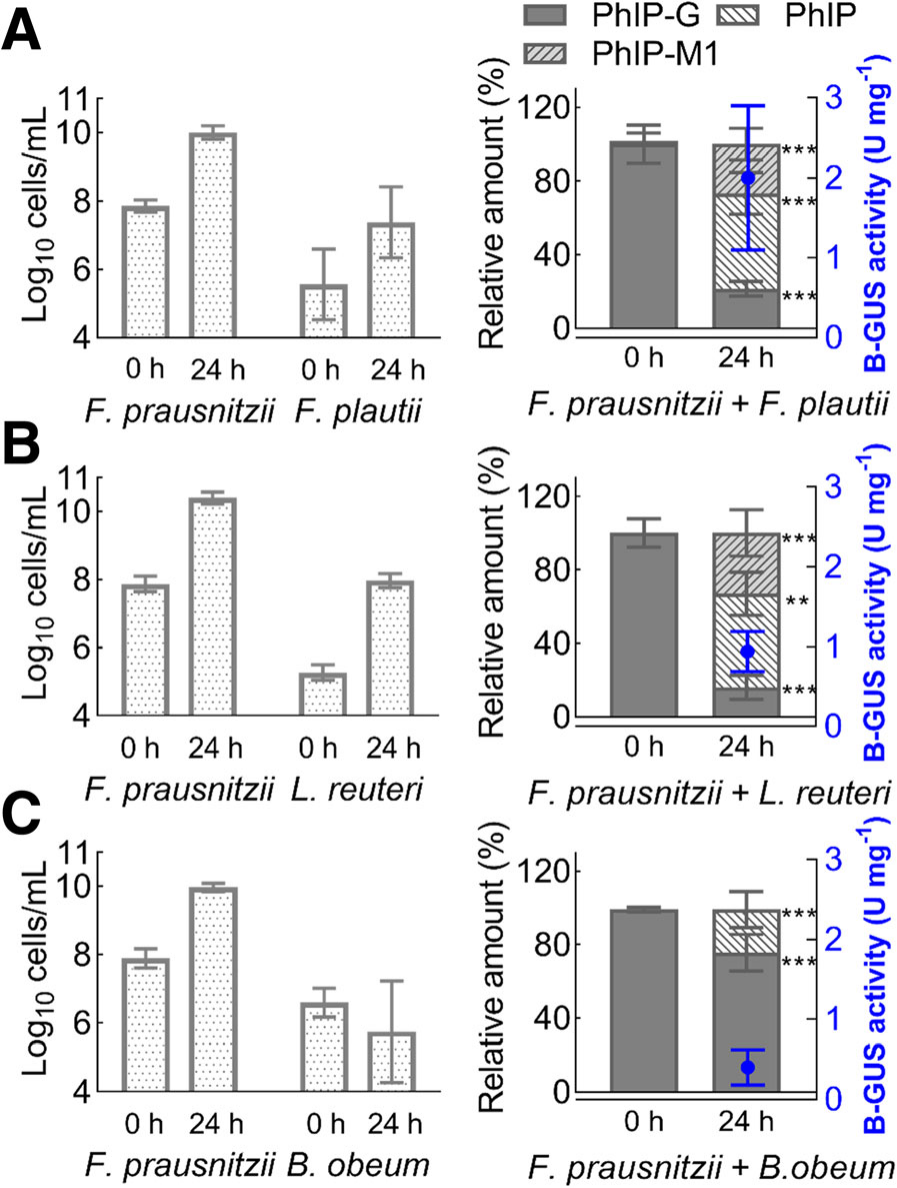
Transformation of PhIP-G to PhIP and PhIP-M1 by co-cultures of B-GUS- and GDH-positive strains during growth in YCFA. Transformation of PhIP-G to PhIP and PhIP-M1 by co-cultures of **a** *F. prausnitzii* and *F. plautii*, **b** *F. prausnitzii* and *L. reuteri*, **c** *F. prausnitzii* and *B. obeum* during growth in YCFA with glycerol and glucose (both 50 mM) and PhIP-G (200 nM) at 37 °C for 24 h. PhIP-G, PhIP, and PhIP-M1 were analysed using nanoLC-ESI-MS^2^. Asterisk indicates significant differences on the relative amount of each compound at 24 h and immediately (0 h) after incubation using two-way ANOVA Sidak’s multiple comparisons test: ** *p* < 0.01, *** *p* < 0.001. The B-GUS activity (blue circle) of the bacterial cell extracts was tested with PNP-G

Both *F. prausnitzii* and *F. plautii* grew in co-cultures, as indicated by an increase of cell counts of log 2.1 and 1.8 cells ml^− 1^, respectively. Glucose and glycerol were utilized, and butyrate, formate, and 1,3-PD were produced (Table 3). Moreover, B-GUS activity was higher in co-cultures (2.0 ± 0.9 U mg^− 1^ protein) than in single cultures, and PhIP-G was indeed converted to PhIP (52 ± 11%) and PhIP-M1 (27 ± 8.5%) (Fig. 5a).

**Table 3:** Substrate utilization and metabolite production of co-cultures. Co-cultures were grown in YCFA supplied with glucose and glycerol (both 50 mM) at 37 °C for 24 h. Substrate utilization and metabolite production were quantified with HPLC-RI. *n* = number of biological replicates. Values are presented as mean ± standard deviation

Co-culture^a^	Substrate	n	Substrate utilization (mM)	Metabolite production (mM)				
				Formate	Acetate	Propionate	Butyrate	1,3-PD
*F. prausnitzii* and *F. plautii*	glycerol glucose	4	−11.2 ± 6.6^A*^ − 13.4 ± 4.4^A^	13.1 ± 3.3^A^	−6.7 ± 3.5^A^	− 1.5 ± 1.1^A^	13.3 ± 2.2^A^	1.2 ± 0.4^A^
*F. prausnitzii* and *L. reuteri*	glycerol glucose	3	−26.5 ± 0.6^B^ − 20.6 ± 3.0^B^	10.7 ± 1.9^A^	−2.0 ± 4.9^A^	−1.6 ± 0.5^A^	10.8 ± 1.8^A^	15.9 ± 3.6^B^
*F. prausnitzii* and *B. obeum*	glycerol glucose	2	− 1.8 ± 3.0^B^ − 23.0 ± 1.1^C^	11.5 ± 1.3^A^	14.4 ± 2.8^B^	− 1.6 ± 0.4^A^	9.3 ± 2.8^A^	0^A^

*Different letters represent the significant differences (*p* < 0.05) on the substrate utilization or metabolite production between different co-cultures using multiple comparisons with 2-way ANOVA

When the same experiment was performed with *F. prausnitzii* and *L. reuteri*, growth was again observed (+log 2.5 and 2.7 cells ml^− 1^ during 24 h incubation, respectively), significantly (*p* < 0.05) more glucose and glycerol were used compared to *F. prausnitzii* and *F. plautii*. Butyrate, formate (Table 3), and 1,3-PD were produced, the amount of 1,3-PD was significantly higher (*p* < 0.05) compared to the *F. prausnitzii* and *F. plautii* co-culture. B-GUS activity was lower (0.94 ± 0.25 U mg^− 1^ protein) compared to the co-culture of *F. prausnitzii* and *F. plautii*. PhIP-G was converted to PhIP (51 ± 12%) and PhIP-M1 (33 ± 12%) (Fig. 5b).

A third co-culture evaluated included *F. prausnitzii* and *B. obeum*. Only *F. prausnitzii* grew (+log 2.1 cells ml^− 1^), utilizing glucose to produce formate, acetate, and butyrate (Table 3). There was lower B-GUS activity (0.40 ± 0.22 U mg^− 1^ protein) compared to the other two co-cultures, and PhIP-G was converted only to PhIP (24.0 ± 9.9%) (Fig. 5c). Glycerol was not used and 1,3-PD and PhIP-M1 were not detected consistent with a lack of growth of *B. obeum* (Fig. 5c, Table 3). In co-cultures of *E. eligens* and *B. obeum*, no PhIP-G was hydrolyzed and no PhIP and PhIP-M1 were observed (data not shown), which is in agreement with the comparatively lower B-GUS activity (0.024 ± 0.004 U mg^− 1^ protein) than *F. prausnitzii*.

**Fig. 6:**
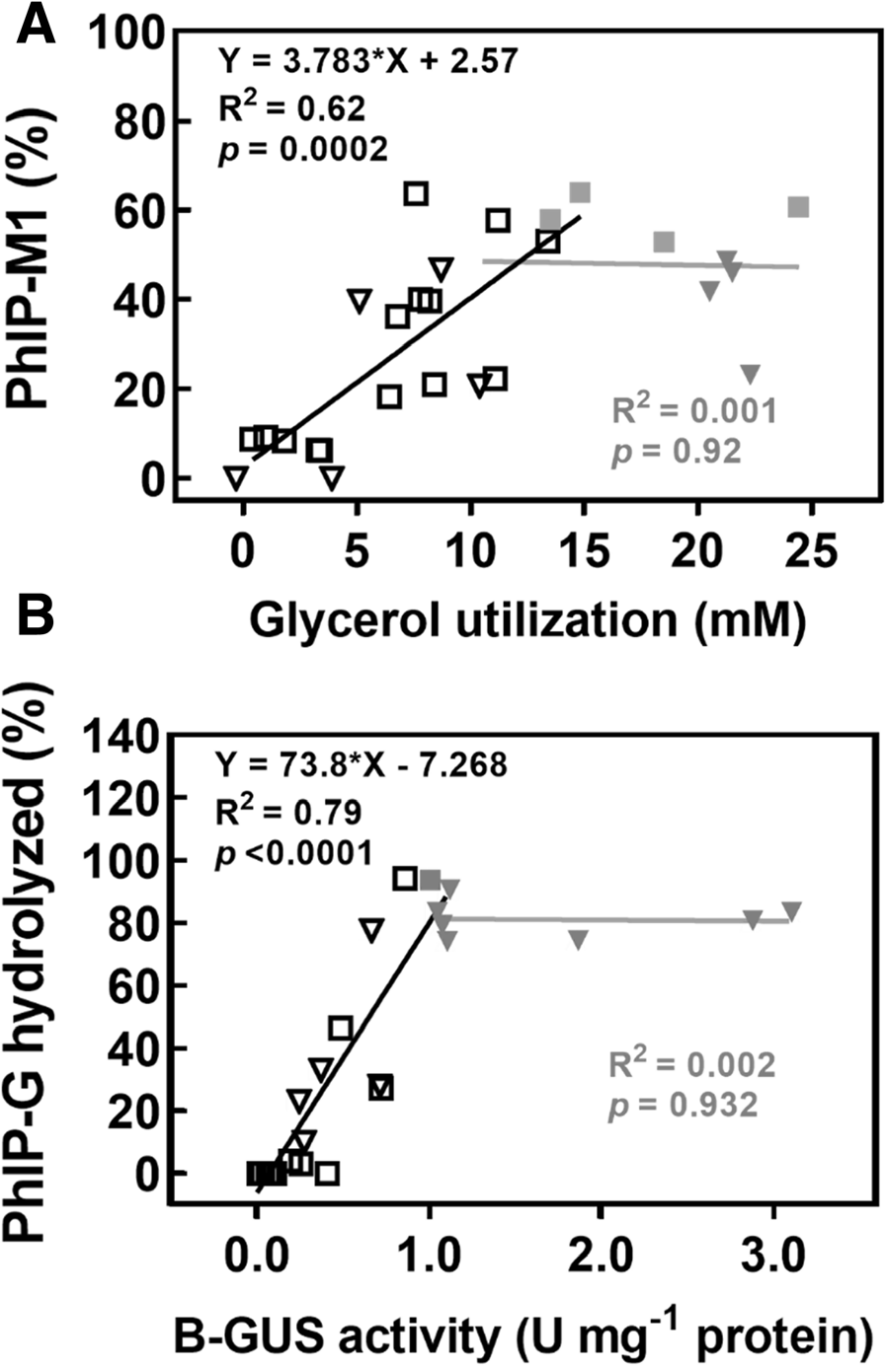
Correlation of glycerol utilization and B-GUS activity with relative PhIP-M1 formation and PhIP-G hydrolysis. **a** correlation of glycerol utilization and PhIP-M1 formation. The proportion of PhIP-M1 was calculated as the ratio of PhIP-M1 relative to the total amount of PhIP and PhIP-M1, **b** correlation of B-GUS activity (determined using PNP-G) and PhIP-G hydrolysis. Data were combined from single cultures (filled and open square) and co-cultures (filled and open triangle). Grey and black symbols in (**a**) represent glycerol utilization above and below 14 mM, respectively. Grey and black symbols in (**b**) represent B-GUS activity above and below 1.0 U/mg protein, respectively

PhIP-M1 formation was correlated in a positive linear relationship with glycerol utilization up to 14 mM in both single and co-cultures (Fig. 6a). At higher concentrations, the correlation plateaued. Similarly, PhIP-G hydrolysis linearly and positively correlated with B-GUS activity and plateaued at approximately 1.0 U mg^− 1^ protein (Fig. 6b). Taken together, co-cultures of *F. prausnitzii* and the GDH positive *F. plautii* and *L. reuteri* were capable of catalysing the two-step process involving the release of PhIP from PhIP-G and its conjugation to form PhIP-M1.

### Fecal microbiome potential to hydrolyse and convert PhIP-G

**Fig. 7:**
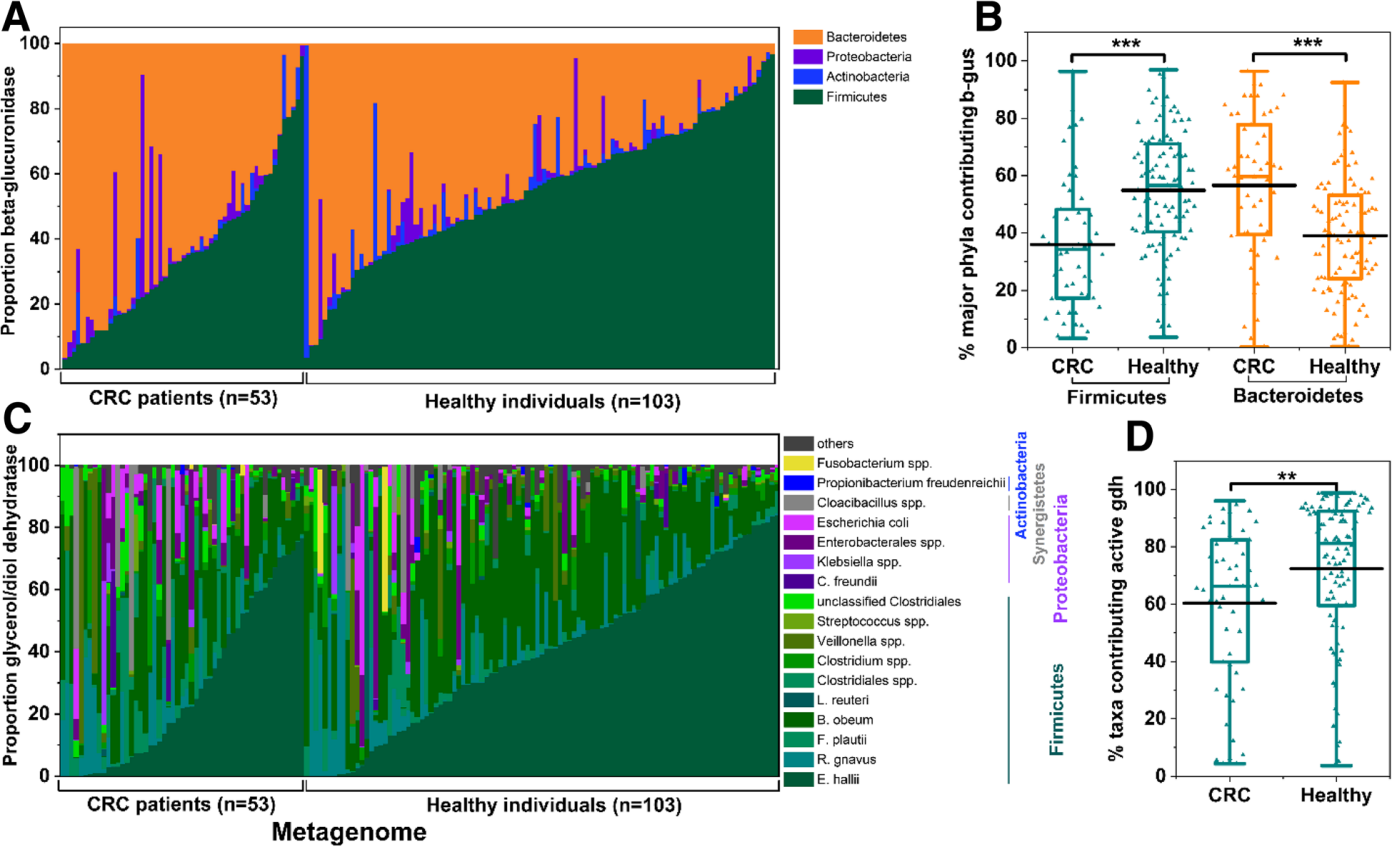
Taxa contributing *b-gus* and *gdh* in fecal metagenomes of healthy individuals and CRC patients*.* Relative abundance of (**a**) phyla contributing *b-gus*, **b** proportion of *Bacteroidetes* and *Firmicutes b-gus* in healthy individuals and CRC patients (taxa contributing *b-gus* are listed in Additional file 1: Table S6), **c** taxa contributing *gdh* to the metagenomes of CRC patients and healthy individuals, **d** proportion of *gdh* of taxa with representatives *E. hallii, B. obeum, F. plautii,* and *L. reuteri* transforming PhIP in the presence of glycerol. Others, groups all taxa with low hits. ** indicates a significant difference at *p* < 0.01; *** indicates a significant difference at *p* < 0.001 with unpaired t-test

To investigate the potential of fecal microbiomes to convert PhIP-G to PhIP-M1, we screened metagenomes of healthy individuals and CRC patients (healthy, *n* = 103, CRC state I-IV, *n* = 53) of a French cohort [25] for *b-gus* and *gdh* using the extensive, generic protein database RefSeq as reference*.* All 156 metagenomes harboured sequence homologous of *b-gus* and *gdh* (Fig. 7**)***.* Mean gene abundance of *b-gus* did not differ between healthy donors (mean 192.6 gene copies per thousand bacterial cells (GC), median: 181.2 GC) and CRC patients (mean 197.2 GC, median: 176.8 GC), but proportions of the main contributing phyla were significantly (*p* < 0.05) different between healthy donors (*Firmicutes* mean: 57.6%, median: 59.5%, and *Bacteroidetes* mean: 39.0%, median: 39.0%) and CRC patients (*Firmicutes* mean: 37.2%, median: 35.5%, and *Bacteroidetes* mean: 56.4%, median: 59.7%) (Fig. 7a, b**)**.

Likewise, mean gene abundance of *gdh* was not different between healthy donors (mean: 57.0 GC, median: 36.3 GC) and CRC patients (mean: 61.4 GC, median: 31.5 GC). For *gdh*, *E. hallii* contributed the highest proportion (mean 28.1%)*,* followed by *B. obeum* (mean 24.5%)*, R. gnavus* (mean: 11.0%) and *F. plautii* (mean: 6.3%) (Fig. 7c)*.* Our activity screening covered representatives of 78% of all *gdh* identified. The proportion of *gdh* of taxa with confirmed HCA transformation (*E. hallii, F. plauttii, B. obeum* and *L. reuteri*) was significantly higher in healthy donors (mean: 72.3%) than in CRC patients (mean: 62.3%, Fig. 7d). This reduction of *gdh* of taxa with confirmed HCA transformation was observed despite converging shifts of *E. hallii* and *F. plautii.* In CRC patients, the proportion of *E. hallii gdh* (mean 28.6%, median: 22.1%) was significantly (*p* < 0.05) lower compared to healthy donors (mean 39.6%, median: 39.4%), whereas the relative abundance of *F. plautii gdh* (mean 8.6%, median: 3.8%) was significantly (*p* < 0.05) increased (mean 3.7%, median: 1.1%, in healthy patients). These data indicate the potential to conjugate the glycerol metabolite acrolein with HCA may be reduced in CRC patients.

## Discussion

In addition to direct physical binding, the interaction of gut microbiota and chemicals including HCA can result in a variety of products with altered bioactivities. Therefore, human gut microbiota is emerging as a decisive modulator of disease risk. In this study, we identified a strain of *F. prausnitzii* to hydrolyze PhIP-G and liberate PhIP (Fig. 1). We newly identified *F. plautii* and *B. obeum* as gut microbes able to convert PhIP to PhIP-M1. Cooperation of *F. prausnitzii* DSM 17677 with strains of *F. plautii* or *L. reuteri* were capable of converting up to one third of PhIP-G all the way to PhIP-M1. Metagenomic mining of 156 fecal microbiomes of healthy individuals and CRC patients revealed that B-GUS is approximately 10 times more abundant than GDH, and that in CRC patients, the proportion of taxa with the capacity to transform PhIP due to GDH activity decreased.

### Role of B-GUS in the release of PhIP from PhIP-G

Gut microbial enzymatic activity has been linked to intestinal diseases and drug-induced gastrointestinal disorders [30–32], and in particular, B-GUS activity has been associated with CRC development [33, 34]. Here we show that PhIP-G, a physiologically relevant and secreted inactive metabolite of the food carcinogen PhIP, can be converted to PhIP by a strain of *F. prausnitzii* (Fig. 1).

In addition to *F. prausnitzii* DSM 17677, we confirmed that *R. hominis* DSM 16839 and *R. intestinalis* DSM 14610 have, albeit lower, B-GUS activity using PNP-G as a substrate [26]. It was previously shown that expression of B-GUS is inducible for some strains, including *R. hominis* DSM 16839 and *E. eligens* DSM 3376 [26, 27]. Inducibility might also be a reason for the low activity observed for *E. eligens* as the purified protein was reported to extensively hydrolyse PNP-G [35].

Despite hydrolyzing PNP-G, neither *Roseburia* strain tested hydrolyzed PhIP-G, possible reasons could be the type of glucuronide linkage and structural differences in B-GUS active sites [29, 36]. PNP-G is an *O*-linked glucuronide, while the glucuronide in PhIP-G is *N*-linked, which might result in a different catalytic efficiency of B-GUS. Indeed, it has been shown that *Escherichia coli* B-GUS preferred *O*-linked glucuronides over *N*-linked glucuronides [36]. PhIP-G is the second most abundant *N*-linked PhIP glucuronide conjugate in humans after OH-*N*^2^-PhIP-G [12]. As both PhIP glucuronide conjugates are *N*-linked, B-GUS of *F. prausnitzii* DSM 17677 would likely hydrolyze OH-*N*^2^-PhIP-G. In agreement, Alexander et al. showed that strains of *E. coli*, *K. pneumoniae*, and *Enterobacter aerogenes* hydrolyzed OH-*N*^*2*^-PhIP-G to release OH-*N*^*2*^-PhIP, and reduced OH-*N*^*2*^-PhIP to PhIP [9].

B-GUS activity was necessary for genotoxicity of 2-amino-3-methyl-3*H*-imidazo [4,5-*f*]quinolone (IQ), another HCA found in cooked meat, in the colon of rats monocolonized with B-GUS-deficient and wild type *E. coli* [37]. The positive correlation observed in the present study between B-GUS activity and PhIP-G hydrolysis, suggests increased PhIP exposure and residence for microbiota with higher B-GUS activity (Fig. 1).

### Cooperation of gut microbes to hydrolyse PhIP-G and conjugate PhIP

Spatial confinement of relevant enzymatic activities, and substrate competition may determine the fate of PhIP-G in the microbial community. Pollet et al. predicted that many *Firmicutes* B-GUS proteins are likely located intracellularly [29], meaning, that in order for PhIP-G as a viable precursor to PhIP, it needs to be transported into the bacterial cell to release PhIP. GDH is also located in the cytoplasm.

Under the conditions used here, concurrent release of PhIP from PhIP-G and its conversion to PhIP-M1 in the presence of *F. prausnitzii* and *L. reuteri* or *F. plautii* led to the formation of approximately 50% PhIP and 30% PhIP-M1 based on original PhIP-G levels, suggesting that bacterial interactions have the capacity to cause PhIP-G to re-enter enterohepatic circulation as well as to block the further mutagenic potential of the resulting PhIP via conversion to PhIP-M1. In a complex gut environment, proportions of PhIP and PhIP-M1 formed from PhIP-G are anticipated to vary depending on microbiota composition, the presence of strains with active B-GUS and GDH, and the availability of glycerol. The reason for *K. pneumonia* not transforming PhIP to PhIP-M1 might be because this species also metabolizes glycerol by an oxidative pathway to finally form dihydroxyacetone phosphate (DHAP), which feeds into glycolysis [38, 39].

### Potential of gut microbial communities to convert PhIP-G to PhIP-M1

The *b-gus* was recently identified as a regular component of fecal metagenomes [29], indicating the potential to hydrolyze glucuronated HCA. Here we confirm that all 156 metagenomes from healthy individuals and CRC patients possessed *b-gus*, suggesting a global potential for HCA-G reactivation. Gene abundance of *b-gus* was on average 12.4 (median 5.6) and 9.7 (median 5.2) fold higher than of *gdh* in healthy donors and CRC patients, respectively.

Compared to *b-gus* [29], there is less known concerning the presence of *gdh* in fecal metagenomes. We observed previously that out of 10 metagenomes, all had a diverse community of strains contributing *gdh* [24], and all of the 156 fecal metagenomes analyzed here harbored *gdh.* Both studies assigned most of the *gdh* to *E. hallii, B. obeum* and *R. gnavus*. Consistently, fecal microbiota from 18 different individuals all possessed GDH activity indicated by PhIP transformation, and the activity greatly differed between individuals (1.8–96%) [17].

Taken together, these data show that B-GUS and GDH are both constituents of the fecal microbiota, and that intestinal microbiota has the potential to both release PhIP from PhIP-G and catalyse its conversion to PhIP-M1, which effectively blocks the bioactivation of PhIP required to induce mutagenicity. The disposition of microbial metabolites of PhIP-G and PhIP may differ in healthy humans versus CRC patients on the basis of changes in the balance of metabolic potential for the hydrolysis and dehydratase processes. While mean gene abundance for each *b-gus* and *gdh* was similar in both groups, we observed that the proportion of contributing bacterial taxa differed in regard to *b-gus* and *gdh.* Relative abundance of *Bacteroidetes b-gus* increased in CRC, and *Bacteroidetes* were reported to mostly carry the BG-type of B-GUS [27, 40]. However, PNP-G activity and B-GUS inducibility differed in a strain-dependent manner within a panel of members of the *Bacteroidetes* and *Firmicutes* [26, 27], therefore it would be speculative to predict B-GUS activity based on the metagenome analysis presented here. Previous studies reported higher fecal B-GUS activity in CRC patients [34], however, B-GUS activity was determined using PNP-G and not HCA-G. There is no literature available concerning the hydrolysis activity of HCA-G of *Bacteroidetes* B-GUS suggesting that further research linking B-GUS diversity, activity and health status is stimulated by the findings presented here. Finally, the proportion of taxa that we could confirm in actively transforming PhIP to PhIP-M1 was lower in CRC than in healthy individuals. This could indeed indicate a reduced potential of the gut microbiota of CRC patients to detoxify HCA.

### HCA transformation versus acrolein exposure

The formation of HCA-M1 from HCA appears to block the potential to activate it to a DNA-reactive species, consistent with data from in vitro mutagenicity studies of these compounds using activating enzymes [17, 20]. Their relative capacities to impact cell viability has also been evaluated to address whether conversion to M1 may increase cytotoxicity. Results were mixed depending on HCA and type of cells. While HCA-M1 cytotoxicity to colon epithelium cannot be excluded, the high concentrations required to reduce cell viability are not consistent with this being a process of significant concern specifically in the context of addressing HCA mutagenicity and carcinogenesis [20, 21, 41]. Nonetheless, the HCA to HCA-M1 conversion process does involve the intermediate formation of acrolein [18, 20], and we recently proposed bacterial glycerol metabolism as a new endogenous source of acrolein [42]. Acrolein is an unspecific antimicrobial agent, which may influence gut microbial composition as some bacteria are more sensitive to acrolein than other taxa [18]. In addition, acrolein can cause oxidative stress and disrupt cell homeostasis in colon epithelial cells [42]. The rate of PhIP-M1 formation is proportional to acrolein concentration [18], suggesting a requirement for relatively high acrolein levels. In agreement, physiologically based pharmacokinetic modelling suggested that high levels of acrolein are required to alter the systemic exposure of the HCA MeIQx in human without changing the intestinal transport [43]. As acrolein itself is toxic and can be endogenous or exogenous [42, 44], further research is necessary to evaluate the toxicological relevance and overall impact on human health from microbial mediated shifts in the disposition of HCA-G, HCA, HCA-M1 and acrolein in the intestine.

## Conclusion

There is a number of reports on the association of gut microbiota dysbiosis and CRC development, but little information on the role of specific bacterial metabolic activities and interaction with dietary compounds. Gut microbes processing GDH activity have the capacity to transform HCA, however, major hepatic metabolites are HCA-G, suggesting critical evaluation of HCA-G transformation is needed. Here we confirmed that gut microbes exerting B-GUS activity have the ability to hydrolyse PhIP-G to release PhIP and, those exerting GDH activity can transform free PhIP to PhIP-M1 in cooperation. Concurrent activity of B-GUS and GDH may lead to formation of a product with reduced mutagenic potential, however, further investigations are needed to evaluate how bacterial reactions of PhIP-G and PhIP impact health, particularly considering the intermediacy of acrolein in this process As a first step toward addressing the relevance of these activities in humans, metagenomic mining confirmed the potential of the human gut microbiome to encode B-GUS and GDH activity. These results are the first observation that the bacterial B-GUS and GDH cooperatively mediate the stepwise conversion of HCA-G to HCA-M1 via intermediate HCA, and provide potential targets to modulate gut microbial activities for mitigating the risk of HCA carcinogenesis.

## Methods

### Strains and culture conditions

All the strains were obtained from the Deutsche Sammlung von Mikroorganismen und Zellkulturen GmbH (DSMZ, Braunschweig, Germany) or from the strain collection of the ETH Laboratory of Food Biotechnology (Table 1).

Bacteria were reactivated from a − 80 °C stored glycerol stock and routinely cultivated in Hungate tubes using anaerobically prepared, modified YCFA medium containing 50 mM glucose (Additional file 1: Table S4 as described previously [28].

### Chemicals

PhIP and PhIP-*N*^*2*^*-β-*D*-*glucuronide (PhIP-G) were purchased from Toronto Research Chemicals (North York, Canada). All other chemicals are listed in Additional file 1: Table S5. PhIP and PhIP-G were applied at 200 nM which is a physiological relevant concentration considering a daily of PhIP ranging from 72 ng [45] to 5000 ng [15] and a colonic volume of 160 to 203 ml in healthy adult humans [46].

### Bacterial cultivation

For growth assays, 2% of an overnight culture was inoculated into 10 ml anaerobically prepared YCFA medium containing glucose, glycerol, 1,2-PD, or glucose and glycerol (all 50 mM) in Hungate tubes. Optical density at 600 nm was monitored immediately and 2, 4, 6, 8, and 24 h after inoculation using a WPA CO 8000 Cell Density Meter (BIOLABO Scientific Instruments, Châtel-St-Denis, Switzerland). Incubation was at 37 °C without shaking.

To test the transformation capacity of single strains, PhIP or PhIP-G (each 200 nM) was added and mixed thoroughly with 10 ml YCFA medium containing glycerol or glycerol and glucose, respectively, before addition of an overnight culture (2%). Samples (1 ml) were transferred into a 1.5-ml tube, immediately (t = 0 h) or after 24 h incubation at 37 °C, centrifuged (20,800 rcf × 5 min), and supernatant was transferred to a new 1.5-ml tube. Supernatants and cell pellets were stored at − 20 °C until further analysis. The experimental procedure for the co-culture study was exactly as described above, except that 1% (0.1 ml) of overnight culture of each bacterium was inoculated into YCFA containing glucose and glycerol. All experiments were carried out three times unless otherwise indicated.

### Analysis of substrate consumption and metabolite formation

Glucose, glycerol, 1,2-PD, 1,3-PD, formate, acetate, propionate, and butyrate were quantified with HPLC-RI using external standards [28]. Supernatants were diluted 1:1 with ddH_2_O. Analytes were separated on an Aminex HPX-87H column (300 × 7.8 mm, 9 μm particle size; Bio-Rad Laboratories AG, Cressier, Switzerland) operated at 40 °C using isocratic conditions (10 mM H_2_SO_4_; 0.4 ml min^− 1^). The injection volume was 40 μl. Detection limits were 1 mM for glucose, 0.9 mM for glycerol, 0.2 mM for 1,2-PD and 1,3-PD, and 0.5 mM for formate, acetate, propionate and butyrate.

### Analysis of B-GUS activity of crude cell extracts

B-GUS activity of crude cell extracts was tested using *para*-nitrophenol-*O*-*β*-D-glucuronide (PNP-G) as a substrate. For cell extract preparation, pellets of 2 mL culture grown for 24 h in YCFA with 50 mM glycerol were re-suspended in 100 μl sodium phosphate buffer (100 mM, pH 6.5) in a Lysing Matrix E tube (MP Biomedicals, Solon, Switzerland). Cells were disrupted using a FastPrep (MP Biomedicals) for 40 s at 6 m s^− 1^ and centrifuged (16,900 rcf × 5 min). Supernatants were used for analysis. Cell extract (5%) was mixed with sodium phosphate buffer containing PNP-G (10 mM) in a 96-well plate. Absorbance (405 nm) was recorded immediately and after 1 h incubation at 37 °C to determine the PNP released in reference to an external calibration curve. Protein concentration in the cell extract was determined with a Bradford protein assay [47].

### DNA isolation and qPCR

Genomic DNA was isolated from 0.5 ml culture using the FastDNA SPIN Kit for Soil (MP Biomedicals). 16S rRNA gene counts were determined by qPCR using primers targeting 16S rRNA of *F. prausnitzii*, *B. obeum*, *E. hallii*, or *gdh* of *L. reuteri*, and *F. plautii* (Additional file 1: Table S3). Reactions were conducted using a 7500 Fast Real-Time PCR System (Applied Biosystems, Zug, Switzerland) and the Kapa SYBR FAST qPCR Master Mix Kit (Labgene Scientific, Châtel-Saint-Denis, Switzerland). Thermal cycling started with a denaturation step at 95 °C for 3 min, followed by 40 cycles consisting of denaturation (95 °C, 3 s) and combined annealing and extension (60 °C, 30 s), followed by melting curve analysis. Agarose gel electrophoresis was performed to verify the specificity of amplification and amplicon size. Standard curves were prepared from 10-fold dilutions of purified PCR amplicons of the gene of interest. Linear detection range was between log 3 and log 8 gene copies for 16S rRNA gene of *F. prausnitzii*, between log 3 and log 9 gene copies for 16S rRNA gene *B. obeum*, and between log 3 and log 10 for *gdh* of *L. reuteri* and *F. plautii*. A factor of 1 and 6 was used to calculate the number of cells for *F. prausnitzii* [48] and *B. obeum* [49], respectively, to account for several copies of 16S rRNA gene.

### Analysis of PhIP-G, PhIP, and PhIP-M1 by nano flow liquid chromatography electrospray ionization tandem mass spectrometry (nanoLC-ESI-MS^2^)

PhIP-G, PhIP, and PhIP-M1 were quantified by nanospray liquid chromatography equipped with a nano-Acquity Ultra Performance LC system (Waters Corporation, Milford, MA, US) and a TSQ Vantage triple quadruple mass spectrometer (nanoLC-ESI-MS^2^). In brief, 100 μl of supernatant was mixed with the internal standard 2-amino-1-methylbenzimidazole (AMBI, 20 μl of 1 μM) and dried under vacuum (miVac Duo Concentrator, Genevac, Suffolk, UK). The residue was re-dissolved with 3 × 100 μL of mixture of acetonitrile:methanol (1:1), vortexed, and centrifuged (16,900 rcf × 5 min). The resulting supernatants were combined in a new 1.5 ml microcentrifuge tube and vacuum-dried. The residue was dissolved in 10% acetonitrile, filtered (0.22 μm PVDF syringe filter, BGB Analytik USA LLC), and the filtrate was transferred to a LC vial with a 250-μl glass insert.

Analytes were trapped in a trap column (Symmetry C18 Trap column, 5 μm, D × L 180 μm × 20 mm, Waters) at trapping conditions (100% solvent A (H_2_O with 0.1% formic acid), 4 μl min^− 1^, 3 min). The injection volume was 0.2 μl. The trap column was connected to the nano analytic column (HSS T3 column, 1.8 μm, D × L 75 μm × 250 mm, Waters) and compounds were eluted with solvent A and solvent B (acetonitrile containing 0.1% formic acid) using the following gradient at 0.5 μl min^− 1^:0–10% A (0–2 min), 10–70% A (2–20 min), 70–90% A (20–20.5 min), 90% A (20.5–28 min), followed by re-equilibrium. Positive ion spectra were recorded using the following parameters: capillary temperature, 270 °C; spray voltage, 2.1 kV; and S-lens 76 units. Compounds were monitored using the following transitions: PhIP-G, 401➔225 collision energy (CE) 30 eV, 401 ➔ 210 CE 30 eV; PhIP, 225 ➔ 210 CE 29 eV, 225➔ 140 CE 50 eV, 225➔ 115 CE 48 eV; PhIP-M1, 281 ➔ 263 CE 28 eV, 281➔ 225 CE 34 eV, 281 ➔ 210 CE 38 eV; and AMBI, 148 ➔ 133 CE 33 eV. The transitions were selected based on previous studies [16, 50]. The collision energy for each transition was optimized by using a mixture of authentic standards. The analyte response is linear in the range of 1–100 nM for PhIP and PhIP-M1, and 0.5–100 nM for PhIP-G (Additional file 1: Figure S3). System control, data acquisition and processing were performed using Thermo Xcalibur software. The limit of quantification was between 10 nM for PhIP and PhIP-M1 and 1 nM for PhIP-G, respectively, based on signal-to-noise ratio (> 10:1).

### Gene abundance of fecal metagenomes for B-GUS and GDH activities

To investigate the distribution of *b-gus* and *gdh* in fecal metagenomes, we reanalyzed metagenomes of healthy donors (*n* = 103), and of CRC state I-IV patients (*n* = 53) of previously generated datasets from a French cohort [25]. The dereplicated gene catalogue generated in [25] was aligned against the bacterial RefSeq database (Release 85, downloaded at 04.01.2018) [51] using DIAMOND (v0.9.13, BlastX in sensitive mode) [52]. Alignments with a bitScore lower than 99% of the best alignment were removed. Furthermore, alignments with a query and reference coverage < 80% or > 130% were filtered, and those with less than 50% positives were removed. Genes aligning to *b-gus* and *gdh* were extracted, leading to a reduced gene catalogue containing 420 candidate genes. The corresponding B-GUS were assigned to EC 3.2.1.31 while GDH were assigned to EC 4.2.1.28 and 4.2.1.30. Taxonomic annotation of candidates was derived using the RefSeq sequence information. For each metagenome candidate gene abundance was extracted from the gene length normalized gene abundance matrix [25]. Abundance was multiplied with 1000 and further normalized by median marker gene abundance [25, 53] to report gene copies per thousand cells (GC).

### Statistical analysis

Significant differences on the relative amount of PhIP-G, PhIP, and PhIP-M1 at 24 h and immediately after incubation (0 h), as well as substrate utilization and metabolite production were determined using a two-way ANOVA Sidak’s multiple comparisons test. Differences of the mean coding potential of *b-gus* and *gdh* were determined using t-test.

## Additional file

Additional file 1: **Table S1.** Substrate utilization and metabolite production of single strains in the presence of glucose (50 mM) in YCFA medium containing acetate during growth for 24 h. **Table S2.** *β*-Glucuronidase (B-GUS) activity of single strains was tested with the absorbance assay using PNP-G as a probe. **Table S3.** Primers used in this study. **Table S4.** Composition of YCFA medium. **Table S5.** Names, suppliers and identifiers of chemicals, solvents, and materials. **Table S6.** Taxa assigned to the phyla *Bacteroidetes, Firmicutes, Actinobacteria* and *Proteobacteria* contributing *b-gus* to fecal metagenomes of healthy individuals and colorectal cancer patients. **Figure S1.** Chromatograms of PhIP-G to PhIP and PhIP-M1 standard and in fermentation of *F. plautii* at 24 h, which is referred to Fig. 3. **Figure S2.** Chromatograms of PhIP-G to PhIP and PhIP-M1 standard and in fermentation of *F. prausnitzii* at 24 h, which is referred to Fig. 4. **Figure S3.** Representative calibration curves used for quantification of PhIP, PhIP-M1 and PhIP-G. The chemical analog AMBI served as the internal standard. (DOCX 265 kb)
