# Supplementary material for: Gut microbial beta-glucuronidase and glycerol/diol dehydratase activity contribute to dietary heterocyclic amine biotransformation
Source: BMC Microbiol. 2019 May 16;19:99. doi: 10.1186/s12866-019-1483-x (PMC6524314; doi:10.1186/s12866-019-1483-x)
Supplement: Supplementary file 1 — Table S1. Substrate utilization and metabolite production of single strains in the presence of glucose (50 mM) in YCFA medium containing acetate during growth for 24 h. Table S2. β-Glucuronidase (B-GUS) activity of single strains was tested with the absorbance assay using PNP-G as a probe. Table S3. Primers used in this study. Table S4. Composition of YCFA medium. Table S5. Names, suppliers and identifiers of chemicals, solvents, and materials. Table S6. Taxa assigned to the phyla Bacteroidetes, Firmicutes, Actinobacteria and Proteobacteria contributing b-gus to fecal metagenomes of healthy individuals and colorectal cancer patients. Figure S1. Chromatograms of PhIP-G to PhIP and PhIP-M1 standard and in fermentation of F. plautii at 24 h, which is referred to Fig. 3. Figure S2. Chromatograms of PhIP-G to PhIP and PhIP-M1 standard and in fermentation of F. prausnitzii at 24 h, which is referred to Fig. 4. Figure S3. Representative calibration curves used for quantification of PhIP, PhIP-M1 and PhIP-G. The chemical analog AMBI served as the internal standard. (DOCX 265 kb) [file 12866_2019_1483_MOESM1_ESM.docx]

**Additional file 1**

**Gut microbial beta-glucuronidase and glycerol/diol dehydratase activity contribute to dietary heterocyclic amine biotransformation**

Jianbo Zhang^1,3^, Christophe Lacroix^1^, Esther Wortmann^1^,^,^ Hans-Joachim Ruscheweyh^2^, Shinichi Sunagawa^2^, Shana J. Sturla^1^, Clarissa Schwab^1^

^1^ Department of Health Sciences and Technology, ETH Zürich, Zürich, Switzerland

^2^ Department of Biology, ETH Zürich, Zürich, Switzerland

^3^ Current address: Department of Biological Engineering, Massachusetts Institute of Technology, Cambridge, MA

Address correspondence to Clarissa Schwab, clarissa.schwab@hest.ethz.ch.

Table S1. Substrate utilization and metabolite production of single strains in the presence of glucose (50 mM) in YCFA medium containing acetate during growth for 24 h. Values are given as mean ± standard deviation, all experiments were performed at least three time (n ≥ 3) with the exception of *E. hallii* (n=2), n.d. = not determined

| Strain | n | Substrate utilization (mM) | Metabolite production (mM) | | | | |
| --- | --- | --- | --- | --- | --- | --- | --- |
|  |  |  | Formate | Acetate | Propionate | Butyrate | 1,3-PD |
| *B. obeum* | 4 | –31.4 ± 5.2 | 8.7 ± 2.0 | 46.0 ± 5.2 | -1.3 ± 0.3 | -0.3 ± 0.2 | n.d. |
| *E. eligens* | 3 | –25.3±1.4 | 17.9±1.6 | 7.7±1.5 | -0.6±0.4 | 0.7±0.6 | n.d. |
| *E. hallii* | 2 | –41.9 | 23.5 | -20.7 | -5.0 | 43.0 | n.d. |
| *F. prausnitzii* | 3 | –6.9±2.5 | 14.9±2.4 | -4.5±1.5 | -0.4±.01 | 11.9±2.2 | n.d. |
| *F. plautii* | 4 | –1.1 ± 0.9 | 0.0 ± 0.0 | 2.6 ± 0.5 | -0.9 ± 0.4 | 5.4 ± 1.8 | n.d. |
| *I. butyriciproducens* | 4 | –0.4 ± 1.9 | -0.7 ± 0.9 | 3.2 ± 2.8 | 1.1 ± 1.4 | 5.9 ± 1.3 | n.d. |
| *L. reuteri* | 3 | –14.0 ± 4.7 | 0.0 ± 0.0 | 1.1 ± 1.6 | -0.9 ± 0.3 | -0.1 ± 0.1 | n.d. |
| *R. hominis* | 4 | –34.5±3.2 | 5.7±1.4 | -25.6±1.6 | -1.6±0.5 | 33.3±2.4 | n.d. |
| *R. intestinalis* | 3 | –39.0 ± 6.2 | 13.1 ± 1.2 | -19.5 ± 3.2 | -1.1 ± 0.2 | 32.8 ± 3.0 | n.d. |
| *R. gnavus* | 3 | –30.3 ± 1.0 | 24.7 ± 2.1 | 16.8 ± 6.2 | 0.3 ± 1.0 | 0.0 ± 0.0 | n.d. |
| *V. dispar* | 5 | –17.1 ± 9.0 | 2.2 ± 0.5 | -0.4 ± 1.6 | 1.0 ± 1.7 | 0.0 ± 0.0 | n.d. |
| *B. fragilis* | 3 | –11.3 ± 5.0 | 1.6 ±1.5 | 7.0 ± 3.8 | -0.1 ± 1.0 | 0.0 ± 0.0 | n.d. |
| *C. freundii* | 3 | –8.8 ± 2.7 | 9.6 ± 1.3 | 5.3 ± 3.0 | -0.2 ± 0.4 | 0.0 ± 0.0 | n.d. |
| *K. pneumoniae* | 3 | –32.8 ± 5.5 | 17.3 ± 1.3 | 2.4 ± 3.1 | 1.8 ± 0.6 | 0.0 ± 0.0 | n.d. |

Table S2. *β*-Glucuronidase (B-GUS) activity of single strains was tested with the absorbance assay using PNP-G as a probe. The B-GUS activity is given as mean ± standard deviation. ND = not detected, n = number of replicates. The presence of *b-gus* was predicted based on Dabek et al. (1) and McIntosh et al (2).

| Bacterial strain | B-GUS activity [U/mg protein] | n | *b-gus* gene | Type of B-GUS according to McIntosh et al. (2) |
| --- | --- | --- | --- | --- |
| *B. obeum* | ND | 5 | - | - |
| *E. eligens* | 0.024 ± 0.004 | 3 | + | GUS |
| *E. hallii* | ND | 2 | - | - |
| *F. prausnitzii* | 0.732 ± 0.208 | 7 | + | GUS, BG |
| *F. plautii* | ND | 3 | - | - |
| *I. butyriciproducens* | ND | 3 | - | - |
| *L. reuteri* | ND | 1 | - | - |
| *R. hominis* | 0.170 ± 0.198 | 5 | + | No information |
| *R. intestinalis* | 0.87 ± 0.157 | 6 | + | GUS |
| *R. gnavus* | ND | 3 | - | - |
| *V. dispar* | ND | 3 | - | - |
| *B. fragilis* | ND | 3 | + | BG |
| *C. freundii* | ND | 3 | - | - |
| *K. pneumoniae* | ND | 3 | - | - |

Table S3. Primers used in this study

| Primer | Sequence 5′-3′ | Target gene | Source |
| --- | --- | --- | --- |
| Eub 338F  Eub 518R | ACTCCTACGGGAGGCAG ATTACCGCGGCTGCTGG | Total bacteria 16S rRNA | (3) |
| ROB-3  ROB-2 | TGAGGAGACTGCCAGGGA CTCCTTCTTTGCAGTTAGGT | *Blautia obeum*  16S rRNA gene | (4) |
| Fprau 223F  Fprau 420R | GATGGCCTCGCGTCCGATTAG CCGAAGACCTTCTTCCTCC | *Faecalibacterium  prausnitzii*  16S rRNA gene | (5) |
| pduCF  pduCR | CCTGAAGTAAAYCGCATCTT  GAAACYATTTCAGTTTATGG | *Lactobacillus reuteri* *gdh* | (6) |
| Fplau F1  Fplau R1 | CAGTGCCACGTCACCAACG  GCGCACTGGGTCATGACAC | *Flavonifractor plautii gdh* | This study |
| EhalF  EhalR | GCGTAGGTGGCAGTGCAA GCACCGRAGCCTATACGG | *Eubacterium hallii*  16S rRNA gene | (7) |
| GNfor  GNP2mod | TATTTAAAAGGITTYGGIMRICAYGARGA  CCTTCTGTTGTIKBRAARTCIGCRAARTTCCA | *gus* | (1) |
| Ee_B-GUS-F  Ee_B-GUS-R | GAAGAACCATCGGCAGTAC  GAAGCTGTTAGCATGCTGC | *E. eligens b-gus* | This study |
| BGF2  BGR2 | CCGCTGCGCGGIGTIWSIMGICAYCARGA  CGCCCATCGGCGSCRAARTCRAACATRTTCCA | *bg* | (2) |

**Table S4. Composition of YCFA medium**

| **Component** | **Proportion** |
| --- | --- |
| Amicase | 1% (*wt/vol*) |
| Yeast extract | 0.25% (*wt/vol*) |
| Sodium bicarbonate | 0.4% (*wt/vol*) |
| Glucose | 0.9% (*wt/vol*) |
| Mineral solution 1 (0.3% (*wt*/*vol*) Dipotassium phosphate) | 15% (*vol/vol*) |
| Mineral solution 2 (0.3% (*wt*/*vol*) potassium dihydrogen phosphate, 0.6% (*wt/vol*) sodium chloride, 0.6% (*wt/vol*) ammonium sulfate, 0.06% (*wt/vol*) magnesium sulfate, 0.06% (*wt/vol*) calcium chloride) | 15% (*vol/vol*) |
| Vitamin solution (0.01% (*wt/vol*) biotin, 0.01 (*wt/vol*) cobalamin, 0.03% *p*-aminobenzoic acid (*wt/vol*), 0.05% folic acid (*wt/vol*), 0.15% pyridoxamine (*wt/vol*)) | 0.01% (*vol/vol*) |
| Volatile fatty acid mix (32.8% (*vol/vol*) acetic acid, 11.6% (*vol/vol*) propionic acid, 2% (*vol/vol*) isovaleric acid, 1.6% (*vol/vol*) isobutyric acid, 1.8% (*vol/vol*) valeric acid, 50% (*vol/vol*) NaOH 5N | 0.58% (*vol/vol*) |
| Hemin stock solution (50 g L^−1^) | 0.02% (*vol/vol*) |
| Resazurin stock solution (1 mg L^−1^) | 0.1% (*vol/vol*) |
| L-Cysteine hydrochloride monohydrate | 0.1% (*wt/vol*) |

**Table S5. Names, suppliers and identifiers of chemicals, solvents, and materials**

| **Reagent or resource** | **Source** | **Identifier** |
| --- | --- | --- |
| **Sodium phosphate buffer** |  |  |
| Na_2_HPO_4_ | Sigma-Aldrich Chemie GmbH | S0876 |
| NaH_2_PO_4_ | Sigma-Aldrich Chemie GmbH | 71496 |
|  |  |  |
| **HPLC** |  |  |
| H_2_SO_4_ | Sigma-Aldrich Chemie GmbH | 5.43827.0250 |
| Methanesulfonic acid | Sigma-Aldrich Chemie GmbH | 471356 |
|  |  |  |
| **Enzymatic assay** |  |  |
| *p*-Nitrophenyl-*β*-D-glucuronide (PNP-G) | Sigma-Aldrich Chemie GmbH | 73677 |
| *p*-Nitrophenol (PESTANAL®, analytical standard) | Sigma-Aldrich Chemie GmbH | 35836 |
| 4-Methylumbelliferyl-*β*-D-glucuronide hydrate | Sigma-Aldrich Chemie GmbH | M9130 |
|  |  |  |
| **LCMS** |  |  |
| PhIP | Toronto Research Chemicals | A617000 |
| PhIP-*N^2^-β-*D*-*glucuronide (PhIP-G) | Toronto Research Chemicals | A617005 |
| 2-Amino-1-methylbenzimidazole (AMBI) | Sigma-Aldrich | 412546 |
| PhIP-M1 | In-house synthesis | (8) |
| Acetonitrile (gradient grade) | Merck | 100030 |
| Methanol (gradient grade) | Merck | 113351 |
| Formic acid (for LC-MS) | Sigma-Aldrich | 5330020050.00 |
|  |  |  |
| **YCFA medium** |  |  |
| Amicase | Sigma-Aldrich Chemie GmbH | A2427 |
| Yeast Extract, Technical, Bacto™ | Becton Dickinson AG | 288620 |
| NaHCO_3_ | Sigma-Aldrich Chemie GmbH | 13433 |
| Glucose | Sigma-Aldrich Chemie GmbH | 16325 |
| 1,2-Propanediol | Sigma-Aldrich Chemie GmbH | 82280 |
| Glycerol | Thermo Fisher Scientific Acros Organics | 158920010 |
| K_2_HPO_4_ | Sigma-Aldrich Chemie GmbH | P5504 |
| KH_2_PO_4_ | VWR International AG | 26923.298 |
| NaCl | Merck | 106404 |
| (NH_4_)_2_SO_4_ | Sigma-Aldrich Chemie GmbH | A5132 |
| MgSO_4_ | Thermo Fisher Scientific Acros Organics | 196850100 |
| CaCl_2_∙2H_2_O | Merck | 102382 |
| Resazurin (Resazurin sodium salt) | Sigma-Aldrich Chemie GmbH | 199303 |
| Hemin (Hemin from bovine) | Sigma-Aldrich Chemie GmbH | H9039 |
| L-cysteine-HCl Monohydrate | Sigma-Aldrich Chemie GmbH | C7880 |
| Sodium hydroxide, Microprills | Sigma-Aldrich Chemie GmbH | 06306 |
|  |  |  |
| **Vitamin Solution** |  |  |
| Cobalamin | Sigma-Aldrich Chemie GmbH | V2876 |
| Biotin | Sigma-Aldrich Chemie GmbH | 14400 |
| *p*-aminobenzoic acid | Sigma-Aldrich Chemie GmbH | A9878 |
| folic acid | VWR International AG | A2085.0010 |
| Pyridoxamine (Pyridoxamine dihydrochloride) | Sigma-Aldrich Chemie GmbH | P9158 |
|  |  |  |
| **Volatile Fatty Acids (VFA mix)** |  |  |
| Propionic acid 99.5% | Sigma-Aldrich Chemie GmbH | P1386 |
| Acetic acid 99.5% | Sigma-Aldrich Chemie GmbH | 33209 |
| Isovaleric acid 99% | Sigma-Aldrich Chemie GmbH | 129542 |
| Isobutyric acid | Sigma-Aldrich Chemie GmbH | 58360 |
| Valeric acid | VWR International AG | A16238.AE |
|  |  |  |
| **SDS-PAGE** |  |  |
| Tris-HCl | Fluka | 93362 |
| Bromophenol blue | Bio-Rad Laboratories Inc. | 1610404 |
| 7.5% Mini-PROTEAN® TGX™ Precast Protein Gels, 10-well, 30 µl | Bio-Rad Laboratories Inc. | 4561023 |
| 10× Tris/Glycine/SDS | Bio-Rad Laboratories Inc. | 1610732 |
| Prism Ultra Protein Ladder (10-245 kDa) | Abcam | ab116028 |
|  |  |  |
| **Software and Algorithms** |  |  |
| Thermo Xcalibur 2.2 | Thermo Scientific | SP1.48 |
| Chromeleon 7 | ThermoFisher Scientific | Version 7.2 |
| Graphpad prism | Graphpad Software, Inc. | Version 7.02 |
| 7500 Fast System Sequence Detection Software | Applied Biosystems | Version 1.4 |

**Table S6. Taxa assigned to the phyla *Bacteroidetes, Firmicutes, Actinobacteria* and *Proteobacteria* contributing *b-gus* to fecal metagenomes of healthy individuals and colorectal cancer patients.** Shown are mean proportions in all data sets (n=156).

| **Phylum** | **Taxon** | **Mean proportions** |
| --- | --- | --- |
| *Bacteroidetes* | Unclassified *Bacteroidales* | 1.01 |
|  | Unclassified *Bacteroides* | 6.93 |
|  | *B. acifidaciens* | 0.04 |
|  | *B. caccae* | 0.02 |
|  | *B. cellulosilyticus* | 1.30 |
|  | *B. clarus* | 1.04 |
|  | *B. coprocola* | 1.70 |
|  | *B. finegoldii* | 0.85 |
|  | *B. fragilis* | 2.97 |
|  | *B. ihuae* | 0.13 |
|  | *B. intestinalis* | 0.39 |
|  | *B. luti* | 0.17 |
|  | *B. nordii* | 0.04 |
|  | *B. ovatus* | 2.34 |
|  | *B. plebeius* | 2.67 |
|  | *B. salanitronis* | 0.02 |
|  | *B. thetaiotamicron* | 2.70 |
|  | *B. uniformis* | 6.54 |
|  | *B. xylanisolvens* | 0.80 |
|  | Unclassified *Prevotella* | 0.78 |
|  | *P. brevis* | 1.02 |
|  | *P. paludivivens* | 0.04 |
|  | *Paraprevotella clara* | 0.48 |
|  | Unclassified *Parabacteroides* | 2.29 |
|  | *Pa. johnsonii* | 0.17 |
|  | *Pa. merdae* | 1.81 |
|  | Unclassified *Alistipes* | 1.22 |
|  | *A. finegoldii* | 1.85 |
|  | *A. senegalensis* | 0.19 |
|  | *A. shahii* | 6.49 |
|  | *A. timonensis* | 0.16 |
| *Firmicutes* | Unclassified *Firmicutes* | 6.61 |
|  | *Faecalibacterium prausnitzii* | 11.12 |
|  | *Roseburia hominis* | 6.67 |
|  | *R.intestinalis* | 1.24 |
|  | *Eubacterium rectale* | 1.93 |
|  | *E. eligens* | 4.92 |
|  | *E. ramulus* | 1.00 |
|  | Unclassified *Clostridium* | 0.79 |
|  | *C. clostridioforme* | 0.27 |
|  | *C. oryzae* | 0.33 |
|  | *C. termitidis* | 1.72 |
|  | *Hungatella hathewayi* | 0.52 |
|  | *Coprococcus eutactus* | 1.04 |
|  | *Flavonifractor sp.* | 0.15 |
|  | *Blautia sp.* | 0.47 |
|  | *Ruminococcus gnavus* | 1.28 |
|  | *R. bicirculans* | 1.16 |
|  | *Gracilibacillus kekensis* | 2.37 |
|  | *Streptococcus pasteurianus* | 0.29 |
|  | *Cohnella* sp. | 1.39 |
| *Actinobacteria* | *Collinsella* sp. | 0.10 |
|  | *Bifidobacterium longum* | 2.98 |
| *Proteobacteria* | *Escherichia coli* | 3.45 |

Standard: PhIP

Sample: PhIP at 24 h

Sample: PhIP at 0 h

Standard: PhIP-M1

Sample: PhIP-M1 at 24 h

Sample: PhIP-M1 at 0 h

Standard: AMBI

Sample: AMBI at 0 h


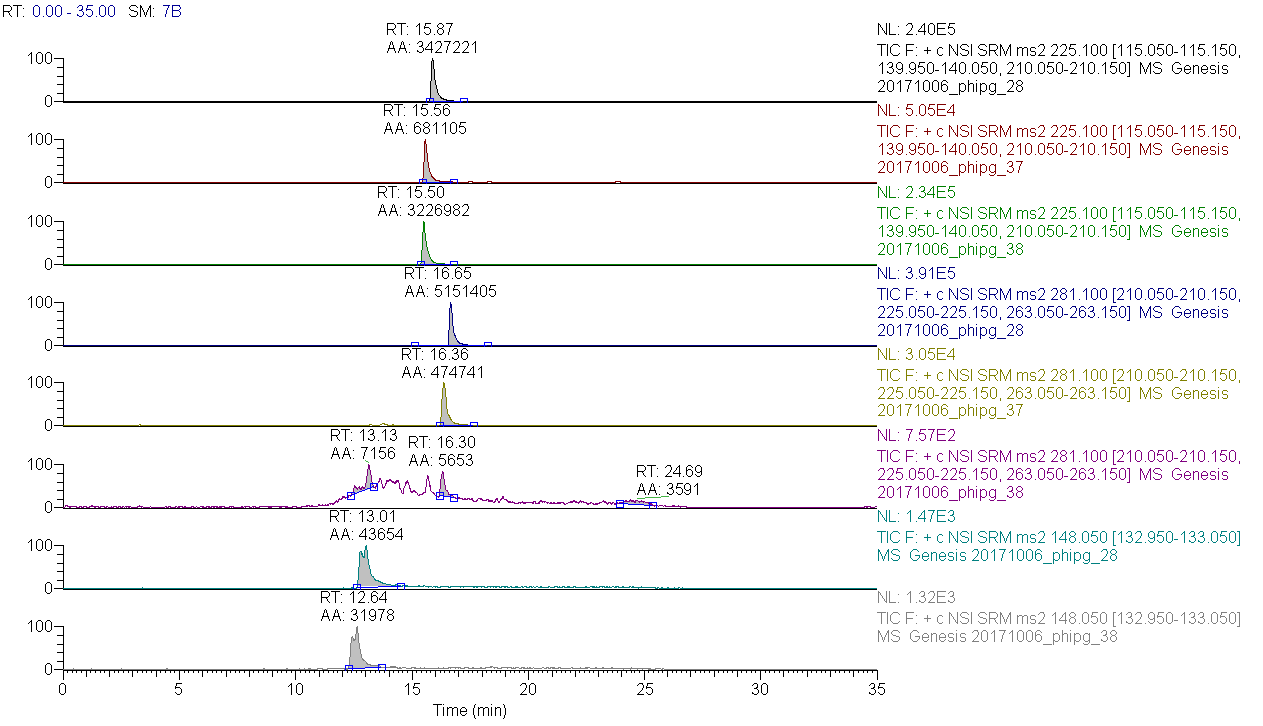


**Figure S1. Chromatograms of PhIP-G to PhIP and PhIP-M1 standard and in fermentation of *F. plautii* at 24 h, which is referred to Figure 3.**


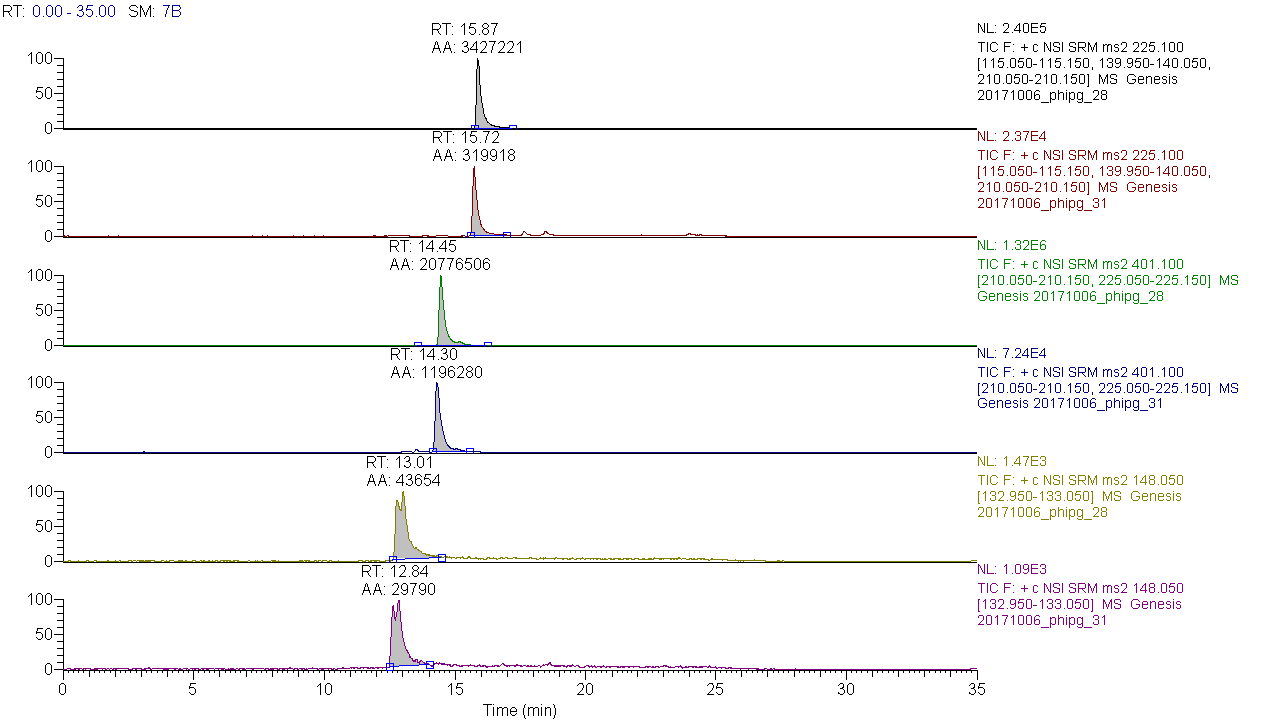


Standard: PhIP

Sample: PhIP at 24 h

Standard: PhIP-G

Sample: PhIP-G at 24 h

Standard: AMBI

Sample: AMBI at 24 h

**Figure S2. Chromatograms of PhIP-G to PhIP and PhIP-M1 standard and in fermentation of *F. prausnitzii* at 24 h, which is referred to Figure 4. The chemical analog AMBI served as the internal standard.**

**Figure S3. Representative calibration curves used for quantification of PhIP, PhIP-M1 and PhIP-G. The chemical analog AMBI served as the internal standard.**

**References**

1. Dabek M, McCrae SI, Stevens VJ, Duncan SH, Louis P. 2008. Distribution of β-glucosidase and β-glucuronidase activity and of β-glucuronidase gene gus in human colonic bacteria. FEMS Microbiol Ecol 66:487–495.

2. McIntosh FM, Maison N, Holtrop G, Young P, Stevens VJ, Ince J, et al. 2012. Phylogenetic distribution of genes encoding β-glucuronidase activity in human colonic bacteria and the impact of diet on faecal glycosidase activities. Environ Microbiol.14:1876–1887.

3. Guo X, Xia X, Tang R, Zhou J, Zhao H, Wang K. 2008. Development of a real-time PCR method for Firmicutes and Bacteroidetes in faeces and its application to quantify intestinal population of obese and lean pigs. Lett Appl Microbiol 47:367–373.

4. Wang RF, Cao WW, Cerniglia CE. 1997. PCR detection of Ruminococcus spp. in human and animal faecal samples. Mol Cell Probes 11:259–265.

5. Bartosch S, Fite A, Macfarlane GT, McMurdo ME. 2004. Characterization of bacterial communities in feces from healthy elderly volunteers and hospitalized elderly patients by using real-time PCR and effects of antibiotic treatment on the fecal microbiota. Appl Environ Microbiol 70:3575–3581.

6. Walter J, Britton RA, Roos S. 2011. Host-microbial symbiosis in the vertebrate gastrointestinal tract and the Lactobacillus reuteri paradigm. Proc Natl Acad Sci 108:4645–4652.

7. Ramirez-Farias C, Slezak K, Fuller Z, Duncan A, Holtrop G, Louis P. 2009. Effect of inulin on the human gut microbiota: stimulation of Bifidobacterium adolescentis and Faecalibacterium prausnitzii. Br J Nutr 101:533.

8. Zhang J, Empl MT, Schwab C, Fekry MI, Engels C, Schneider M, Lacroix C, Steinberg P, Sturla SJ. 2017. Gut microbial transformation of the dietary imidazoquinoxaline mutagen MeIQx reduces its cytotoxic and mutagenic potency. Toxicol Sci 159:266–276.
